# Targeting phosphatidylserine for Cancer therapy: prospects and challenges

**DOI:** 10.7150/thno.45125

**Published:** 2020-07-23

**Authors:** Wenguang Chang, Hongge Fa, Dandan Xiao, Jianxun Wang

**Affiliations:** 1 Institute for Translational Medicine, The Affiliated Hospital, College of medicine, Qingdao University, Qingdao, China.; 2 School of Basic Medical Sciences, College of medicine, Qingdao University, Qingdao, China.

**Keywords:** phosphatidylserine, cancer, T lymphocytes, immunotherapy, bavituximab

## Abstract

Cancer is a leading cause of mortality and morbidity worldwide. Despite major improvements in current therapeutic methods, ideal therapeutic strategies for improved tumor elimination are still lacking. Recently, immunotherapy has attracted much attention, and many immune-active agents have been approved for clinical use alone or in combination with other cancer drugs. However, some patients have a poor response to these agents. New agents and strategies are needed to overcome such deficiencies. Phosphatidylserine (PS) is an essential component of bilayer cell membranes and is normally present in the inner leaflet. In the physiological state, PS exposure on the external leaflet not only acts as an engulfment signal for phagocytosis in apoptotic cells but also participates in blood coagulation, myoblast fusion and immune regulation in nonapoptotic cells. In the tumor microenvironment, PS exposure is significantly increased on the surface of tumor cells or tumor cell-derived microvesicles, which have innate immunosuppressive properties and facilitate tumor growth and metastasis. To date, agents targeting PS have been developed, some of which are under investigation in clinical trials as combination drugs for various cancers. However, controversial results are emerging in laboratory research as well as in clinical trials, and the efficiency of PS-targeting agents remains uncertain. In this review, we summarize recent progress in our understanding of the physiological and pathological roles of PS, with a focus on immune suppressive features. In addition, we discuss current drug developments that are based on PS-targeting strategies in both experimental and clinical studies. We hope to provide a future research direction for the development of new agents for cancer therapy.

## Introduction

Cancer is a leading cause of mortality and morbidity worldwide 1. Currently, the main treatments for cancer are surgery, chemotherapy and radiation therapy, which are designed to eliminate tumor cells by directly removing or killing them 2-4. With extensive research based on tumor biology, various genes and proteins have demonstrated potential inhibitory effects on tumor growth and proliferation, such as noncoding RNA, certain transcription factors and apoptotic pathway activators associated with tumor growth 5-8. Moreover, a new therapeutic strategy, which eliminates tumor cells by enhancing the immunity of patients, called immunotherapy, is becoming a popular therapeutic method for mono- or polytherapy of malignant tumors 9, 10. Immunotherapy fights cancer by helping the immune system recognize and target tumor cells. Until now, several types of immunotherapy have been used for cancer treatment, including T cell transfer therapy, immune checkpoint inhibitors, monoclonal antibodies and vaccines. For instance, chimeric antigen receptor T-cell immunotherapy (CAR-T), a T cell transfer therapy in which T cells from patients are modified and reinfused to better recognize tumor antigens, has been successfully used in some hematologic malignancies 11, 12. Additionally, some agents targeting the immune system have been developed for cancer treatment. Nivolumab and pembrolizumab are immune checkpoint inhibitors that enhance T cell immunity by blocking programmed cell death protein 1 (PD-1). Atezoizumab and avelumab are PD-1 ligand (PD-L1) inhibitors that were approved by the US Food and Drug Administration (FDA) in 2014 for certain cancers 13-16. Alemtuzumab, an antibody that binds to CD52 antigen on lymphocytes and attracts immune cells to destroy these cells, is used in chronic lymphocytic leukemia therapy 17, 18. Nonetheless, antibodies targeting CD47 19, OX40 20, CD20 21 and cytotoxic T-lymphocyte antigen 4 (CTLA-4) all show immune activation effects. However, many patients in the clinic do not respond well 22, 23. For example, some patients have poor responses to PD-L1 inhibitors, which may be due to impaired T cell infiltration *via* upregulation of PD-L1, indoleamine-2,3-dioxygenase (IDO), and FoxP3(+) regulatory T cells (Tregs), termed primary resistance 24 or due to loss of T cell function *via* expression of different immune checkpoint proteins 25, defects in interferon signaling and antigen presentation, termed acquired resistance 26. Thus, new immunostimulatory targets are urgently needed to compensate for the deficiencies in cancer therapy.

Phospholipids compose the asymmetric bilayer membrane in eukaryotic cells 27. Among all the phospholipids, phosphatidylserine (PS) is a negatively charged amino-phospholipid and is predominately localized in the inner membrane leaflet 28. PS exposed on the outer leaflet of the plasma membrane responds to various stimuli; alternatively, PS present in certain vesicle membranes during vesicle generation participates in the progression of various diseases 29-31. In tumor microenvironments, PS exposure on tumor cells and immune cells leads to immune suppression and the promotion of tumor growth. PS exposure on blood cells, microparticles and neutrophil extracellular traps affects procoagulant activity in pancreatic cancer patients 32. Therefore, the location of PS on membranes is important for cell survival, growth, proliferation and cancer-related symptoms 33, 34. In this review, we summarize recent research on the roles of PS in physical and cancer biology, as well as related current clinical pharmacological trials, and we hope to provide new insights into future applications of PS in cancer therapy.

## PS biology

### PS synthesis

**Figure 1:**
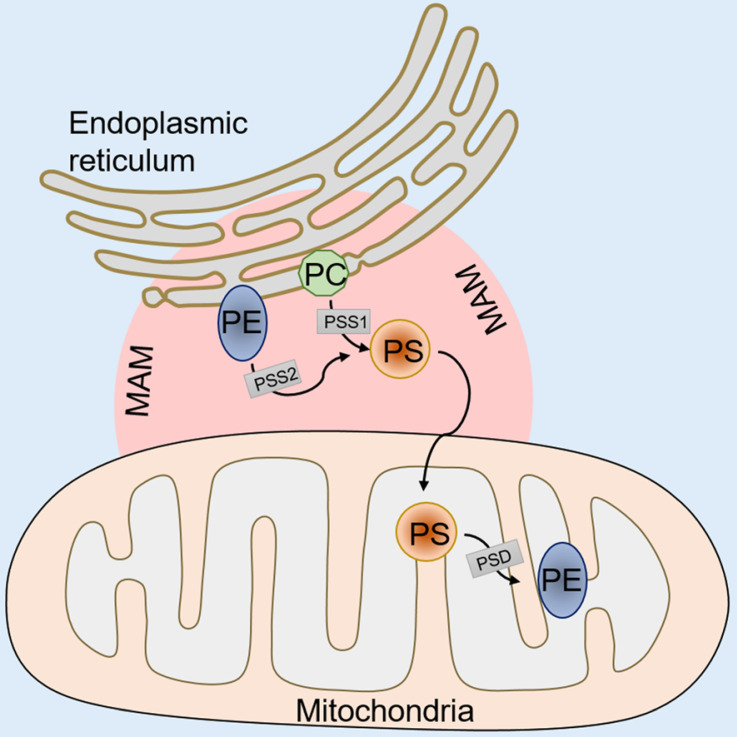
** An illustration of PS synthesis.** PS synthesis in the MAM from PC by PSS-1 or PE by PSS-2. After synthesis, some of the PS transported into mitochondria is decarboxylated to PE by PSD. The remaining synthesized PS in the MAM is transported to other organelles, such as the plasma membrane and the Golgi. MAM, mitochondria associated membranes; PC, phosphatidylcholine; PE, phosphatidylethanolamine; PSS-1/2, phosphatidylserine synthase 1/2; PSD, phosphatidylserine decarboxylase.

In mammalian cells, PS is synthesized in a specific domain of the endoplasmic reticulum called the mitochondria-associated membrane (MAM). The MAM facilitates the molecular exchange between the endoplasmic reticulum and mitochondria, and it plays a pivotal role in maintaining cellular health 35-37. PS synthesis in the MAM is from either phosphatidylcholine (PC) or phosphatidylethanolamine (PE) by phosphatidylserine synthase-1 (PSS-1) (from PC) or phosphatidylserine synthase-2 (PSS-2) (from PE) *via* a base-exchange reaction with serine (**Figure 1**). After synthesis, some of the PS is transported into the mitochondria by physical contact between the MAM and mitochondrial outer membranes 38, 39. Then, the PS in the mitochondria is decarboxylated and PE is synthesized by phosphatidylserine decarboxylase (PSD), an enzyme restricted to the mitochondrial inner membranes (**Figure 1**). This PE synthesis pathway from PS in the mitochondria is essential for the maintenance of mitochondrial integrity and cell growth, and a deficiency in the PSD gene results in embryonic lethality in mice 40, 41. The remaining synthesized PS in the MAM is transported to other organelles, such as the plasma membrane and the Golgi (**Figure 1**). The transportation mechanism is mostly through nonspontaneous diffusion mechanisms, including soluble transport proteins or vesicles 42. The proportion of synthesized PS that enters the mitochondria versus other organelles remains elusive. Previous phospholipid composition analysis of different organelles has shown that the highest PS content can be found in the plasma membrane and the lowest content is in mitochondria in cells of the rat liver and kidney 43, 44. However, the steady state levels of PS may not reflect actual synthesized PS transport because the mitochondrial PS would be rapidly converted to PE by PSD after entering the mitochondria. Additionally, recent studies have shown that the disruption of PS transport to the plasma membrane by knockdown gradient required protein rescue of the mitochondrial deficiency induced by PSS knockdown 45, suggesting a coordinated regulation of PS distribution from the ER into the plasma membrane or mitochondria.

### PS exposure regulation

Flippases and scramblases coordinate the exposure of PS 46. The type IV subfamily of P-type ATPases (P4-ATPase) are eukaryotic flippases that translocate phospholipids from the outer leaflet to the inner leaflet of biological membranes by ATP-dependent active transport 47. The human gene encodes five classes of P4-ATPases: Class 1a (ATP8A1, ATP8A2); Class 1b (ATP8B1, ATP8B2, ATP8B3, ATP8B4); Class 2 (ATP9A, ATP9B); Class 5 (ATP10A, ATP10B, ATP10D); and Class 6 (ATP11A, ATP11B, ATP11C). ATP8A1, ATP8A2, ATP11A and ATP11C are known to flip PS from the exoplasmic to the cytoplasmic leaflet 48-50. Mechanistic studies have shown that the activities of ATP11A and ATP11C are affected by the cytosolic Ca^2+^ flux. In normal growing cells, ATP11A and ATP11C persistently transport PS from outer to inner membranes. When cytosolic Ca^2+^ increases due to various stimuli, such as platelet activation, ATP11A and ATP11C are inactivated, which enables exposure of PS on the cell surface. In addition, CDC50 family proteins (CDC50A, CDC50B, and CDC50C) complexed with P4-ATPase are required for normal flippase activity 51. CDC50A deficiency results in increasing PS exposure in cell membranes. It is worth noting that different variants of ATP11C are not functionally equivalent. ATP11C-a participates in PKC-mediated endocytosis, but not ATP11C-b or ATP11C-c, while ATP11C-b regulates the PS distribution in distinct regions of the polarized cell plasma membrane 52.

Scramblases catalyze another kind of phospholipid movement, which normally occurs in apoptotic cells or activated platelets 53. Scramblases mediate nonspecific, bidirectional, and ATP-independent phospholipid movement. TMEM16 and Xk-related protein 8 (Xkr8) are two proteins that have been identified as scramblases for PS transport. TMEM16 mediates Ca^2+^-activated scrambling activity and catalyzes PS exposure on the platelet surface. Xkr8 has been shown to increase PS exposure in response to apoptotic stimuli such as DNA degradation and oxidative stress. The molecular mechanism of Xkr8 activation depends on either a caspase-dependent signaling pathway 54, 55 or regulation *via* phosphorylation 56. The PS exposure mediated by Xkr8 is slow and irreversible, thus facilitating phagocytosis.

### Apoptotic function of PS

The cells in our body constantly renew each day 57; thus, the “replaced” cells must be cleared out efficiently to prevent inflammation or autoimmunity 58. PS provides a recognizable and distinguished signal for cell phagocytic processes, also known as “apoptosis” 59, 60. Apoptotic cell death can be triggered by the intrinsic (*via* mitochondria) pathway or the extrinsic (*via* cell death factor) pathway. Caspase activation is the common signature of these two apoptotic pathways. Cytochrome C activates caspase 3 or caspase 7, which are released from mitochondria and induce the intrinsic apoptotic pathway. Alternatively, caspase 8 is activated by death factors such as tumor necrosis factor (TNF) and Fas ligand (FasL), subsequently activating caspase 3 and inducing apoptosis *via* the extrinsic pathway 61. PS exposure on the cell surface is a hallmark of apoptosis activation, presenting a recognition signal for macrophage recruitment and cell engulfment 62. Caspase 3 and caspase 7 directly activate Xkr8 by cleavage and execute scrambling activity. Mouse and human cancer cells with repressed Xkr8 expression *via* hypermethylation fail to expose PS during apoptosis 63. In addition to Xkr8, Xkr4 and Xkr9 possess a caspase recognition site and aid in PS exposure during apoptosis 64. Additional studies have shown that a complex consisting of Xkr8 with basigin (BSG) and neuroplastin (NPTN), which are type I membrane proteins of the Ig superfamily, is essential for PS exposure. Cells with a knockdown of BSG and NPTN failed to expose PS in response to apoptotic stimuli, though Xkr8 localized intracellularly 65, suggesting the involvement of a more complicated mechanism in caspase-dependent phospholipid scrambling activity. Conversely, caspases also recognize ATP11C sites and inactivated ATP11C on cell membranes. Mutation of the caspase recognition site of ATP11C leads to no exposure of PS during apoptosis and no engulfment by macrophages, indicating that apoptosis-related PS exposure is coordinated by both scramblase and flippase activity.

### Non-apoptotic functions of PS

#### Coagulation

Blood coagulation consists of initiation, amplification and propagation phases 66. Formation of the prothrombin activator complex initiates coagulation. The process involves the activation of several coagulation cascades, including tissue factor (TF), activated factor VII (FVIIa), activated factors IX and X (FIXa and FXa) and the cofactor activated factor V (FVa) 67. PS present in the exofacial leaflet facilitates assembly of the complex and promotes thrombin formation 68, 69. Specifically, PS interacts with the 9-12-carboxyglutamic acid (Gla) domain at the NH_2_-terminus of the coagulation cascade (FVII, FIX, FX, FII) *via* Ca^2+^ 70, 71. PS exposure on platelets is mediated by TMEM16F activation. TMEM16F exerts its scrambling activity in response to Ca^2+^ and inactivates ATP11A and ATP11C, thereby exposing PS on limited regions of the cell surface 55, 72. However, this exposure of PS is transient, as the PS distribution will resume when the Ca^2+^ concentration returns to a normal level. This may explain why under physiological conditions, the constant flipping of PS prevents cells from being engulfed by macrophages 73.

#### Myoblast fusion

Myotube formation by myoblast fusion is essential for skeletal muscle development and regeneration. PS exposure is an initial fusion signal in myoblasts 74. Studies have shown that ATP11A and CDC50A-mediated PS exposure is required for myotube formation by myoblasts. In addition, the activity of a mechanosensitive Ca^2+^ channel that is predominantly expressed during myotube formation, PIEZO1, is affected by flippase-mediated inward translocation of PS from the cell surface. Ca^2+^ influx *via* PIEZO1 is impaired in CDC50A-deficient or ATP11A-deficient C2C12 cells, while this impairment can be recovered by exogenous expression of CDC50A or a PS flippase 75.

Apoptotic myoblasts also expose PS. The differences between the myoblast fusion-related PS exposure and myoblast apoptosis-related PS exposure are still elusive. One report has shown that differentiating muscle cells appear normal in terms of mitochondria potential and negative caspase 3 protein expression. Additionally, myotube formation and exposure of PS cannot be blocked by the caspase inhibitor zVAD(OMe)-FMK 74. However, other reports show that apoptosis blocked by a caspase inhibitor impaired myoblast fusion. They also found that a fraction of the myoblasts underwent apoptosis during myoblast fusion and that this small fraction induced PS exposure to promote myoblast fusion in the presence of caspase inhibitors 76, 77. How apoptotic myoblasts affect healthy myoblast fusion is still under investigation. Xkr8 may play a central role in myoblast differentiation. Studies have shown that Xkr8 knockdown myoblasts exhibit impaired differentiation and more apoptotic cells during differentiation. Moreover, Xkr8 accelerates myoblast differentiation *via* a mechanism unrelated to PS exposure and caspase-dependent cleavage 78.

#### T lymphocyte activation

Immunotherapies rely on boosting the preexisting or inducing a new tumor-resident T cell pool to eliminate tumor cells. CD4^+^ and CD8^+^ T cells are two types of T cells that are closely related to cancer immunotherapies. CD8^+^ T cells are cytotoxic T cells that are considered major drivers of antitumor immunity, while CD4^+^ T cells, including Th1, Th2, Th17, and Treg cells, play a prominent role in controlling tumor growth 79. PS exposure on T lymphocytes is normally associated with dead cell clearance. However, Elliott et al. reported that a subpopulation of activated/memory CD4^+^ T cells, which express low levels of the RB isoform of CD45 (CD4^+^CD45RB^lo^), express high levels of PS but are not apoptotic. In this study, CD45 expression levels inversely correlated with the proportion of T cells that exposed PS following P2X_7_ stimulation induced by benzoyl ATP (BzATP). This specific PS exposure in a subpopulation of T lymphocytes affected T cell migration, infiltration and rapid inflammation responses 80. Furthermore, human CD8^+^ cytotoxic T lymphocytes (CTLs) with antigen (Ag) recognition-induced PS exposure are also not apoptotic. The exposed PS on Ag-specific T cells is concentrated at the immunologic synapse, and a blockade of PS exposure by the annexin V protein during Ag recognition diminishes cytokine secretion 81, indicating that PS exposure on CTLs is related to cell-cell communication and T cell activation. The mechanism of PS exposure on lymphoma cells is related to TMEM16F activation, and this exposure level is comparable to that observed in apoptotic cells 82, again indicating that PS exposure alone is not sufficient to initiate phagocytosis.

### PS receptors

PS receptors function as mediators to invoke immune suppression 83. Multiple proteins and protein families have been identified as PS receptors, including brain angiogenesis inhibitor 1 (BAI1), stabilin-1/2, integrin, TIM family proteins (T cell/transmembrane, immunoglobulin, and mucin), and TAM family proteins (Tyro3, AXL, and the MerTK receptor tyrosine kinase family). BAI1 84, stabilin-1/2 85 and the TIM family proteins 86 directly bind to PS, while TAM and integrin receptors indirectly bind to PS *via* ligand proteins. TAM receptors bind PS *via* the vitamin K-dependent proteins growth arrest-specific 6 (Gas6) and protein S (Pros1) 87 and integrin receptors bind to PS *via* Mfge8 88. Here, we briefly introduce the most studied PS receptors and their expression on immune cells. For more details on each PS receptor, please see other published reviews 89-92.

#### Stabilin-1/2

**Figure 2:**
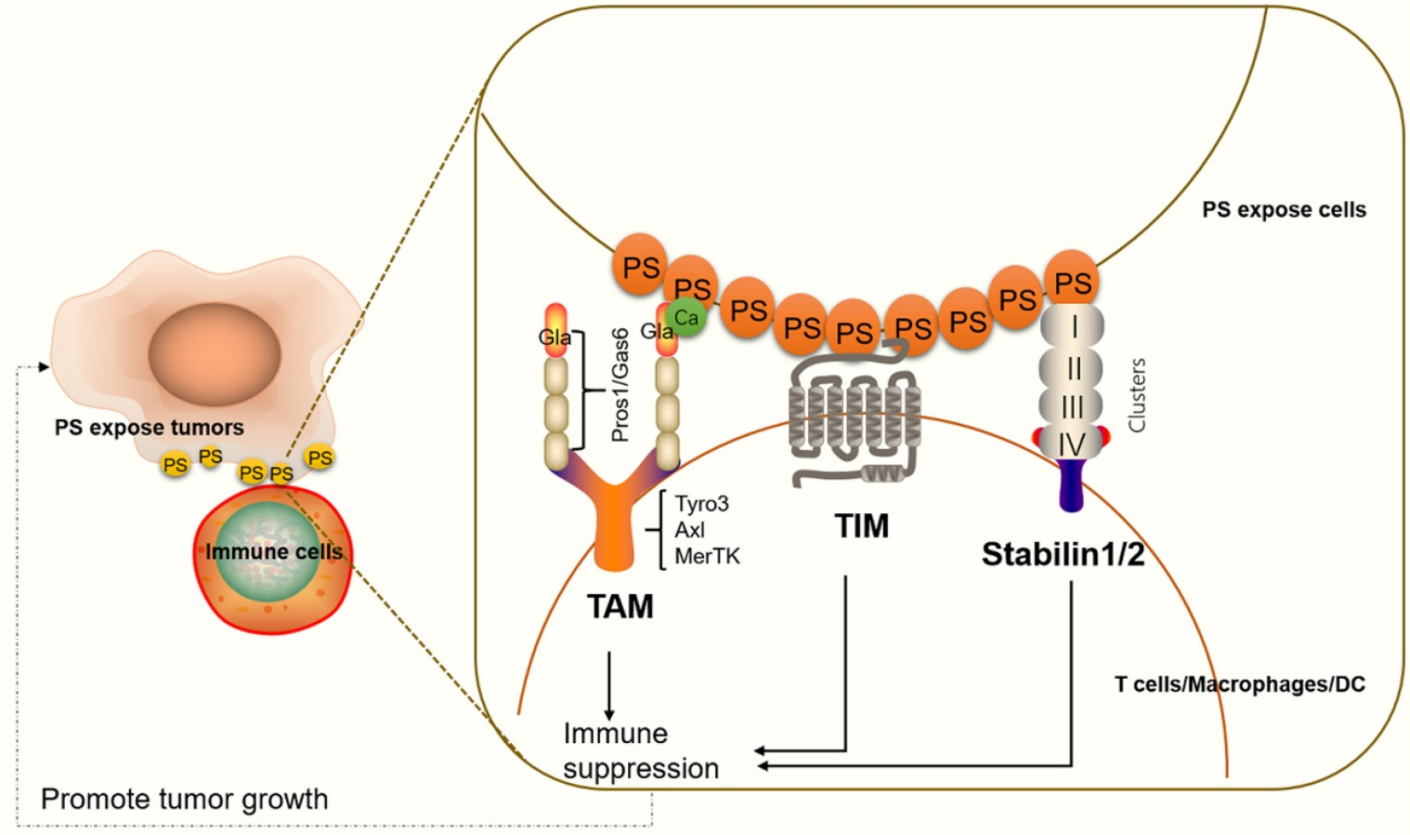
** An illustration of PS receptors and their functions in immune suppression.** TAM interacts with PS through Gla domain-containing proteins, Gas6 or Pros1. Ca^2+^ also participates in effective PS binding and receptor activation. PS binds to TAM to regulate the feedback inhibition of the innate immune response in immune cells. The TIM protein forms a narrow cavity or pocket that PS binds to and plays a critical role in regulating immune responses. Stabilin-1/2 interacts with PS through four clusters (each cluster includes an EGF-like domain, an atypical EGF-like domain, a FAS domain and/or a link domain), which in turn activate a series of signals that lead to immune suppression.

Stabilin-1 and stabilin-2 are widely expressed in endothelial cells in different organs, such as the liver, spleen, lymph nodes, bone marrow (stabilin-2), and adrenal cortex (stabilin-1) 92, 93. In apoptotic cells, stabilin-1 and stabilin-2 directly bind to PS on the cell surface and initiate cell engulfment by activation of Rac family small GTPase 1 (Rac1) *via* a phosphotyrosine-binding domain-containing engulfment adaptor protein (Gulp1)-dependent mechanism or a Gulp1-independent pathway 92. On immune cells, stabilin-1 is expressed in alternatively activated macrophages, also known as M2-like macrophages. M2 macrophages usually activate responses for healing or repair and provide a promoting environment for cancer growth 94, 95. In macrophages cocultured with apoptotic cells, stabilin-1 is recruited to sites of recognition and mediates clearance of dead cell in a PS-dependent manner 85. Additionally, stabilin-1-deficient macrophages in mice show reduced growth of primary tumors compared with controls. Anti-stabilin-1 antibody treatment leads to diminished numbers of immunosuppressive leukocytes in tumors 95, indicating that stabilin-1 on macrophages participates in tumor-related immune responses. Stabilin-2 has also been shown to be expressed on macrophages and to participate in TGF-β production. TGF-β is a key immune suppressive cytokine, which controls the adaptive and innate immune systems by regulating the function and generation of immune cells 96, 97 (**Figure 2**).

#### TIM family

TIM proteins are type I cell surface glycoproteins and share common structural features, including Ig-like, mucin, transmembrane, and cytoplasmic domains 98. Structural studies have shown that PS binds to a narrow cavity formed by the CC and FG loops of IgV domains 99. TIM-1, TIM-3, and TIM-4 belong to the human TIM family and have been identified as PS receptors 100. TIM proteins are expressed on various immune cells and play critical roles in regulating immune responses and viral infections 101, 102. TIM-1 is expressed on CD4^+^ T cells 103, mast cells 104 and regulatory B cells 105. TIM-1 on Th2 cells functions as a potent costimulatory molecule for T cell activation 99. TIM-1-deficient B cells promote Th1 and Th17 responses but inhibit the generation of regulatory T cells 105. Moreover, TIM-1 is a binding site for Ebola virus on T lymphocytes and blocking TIM1-PS interactions reduces viral binding, T-cell activation, and cytokine production 106. TIM-4 is highly expressed on dendritic cells and macrophages, and TIM-4 on macrophages participates in inflammatory responses 107. TIM-3 is not expressed on naïve T cells, but it is expressed on fully differentiated Th1 cells 108. TIM-3 is also expressed on cytotoxic CD8^+^ T cells, Th2 cells, Th17 cells and regulatory T cells 109, 110, as well as on dendritic cells (DCs) and a subpopulation of macrophages 111. Targeting TIM-3 is known to suppress tumor growth (**Figure 2**). We will discuss these details in the section “*Targeting PS receptors and cancer therapy*”.

#### TAM family

The TAM family members indirectly bind to PS *via* the Gla domain of Gas6 or Pros1 ligand. Previous research has shown that Gas6 binds to and activates all three TAM receptors (Tyro3, AXL, and MerTK), while Pros1 is a ligand only for Tyro3 and MerTK 112. However, recent evidence has shown that Pros1 does bind to and activate AXL in glioma sphere cultures 113, and it modulates AXL expression in oral squamous cell carcinoma cell lines 114, suggesting that Pros1 induces receptor activity function differently in different tumor microenvironments. Of all three receptors, MerTK is the most studied receptor regarding immune responses. MerTK is expressed on dendritic cells, nature killer cells, B cells, and macrophages 115, 116. Regarding T cells, MerTK is expressed both on CD4^+^ T cells and CD8^+^ T cells. MerTK on dendritic cells competes for Pros1 interaction with MerTK in CD4^+^ T cells to control T cell activation 117 (**Figure 2**).

## PS as an immune therapy target for cancer

### PS exposure on tumor cells and induction of immune suppression

**Figure 3:**
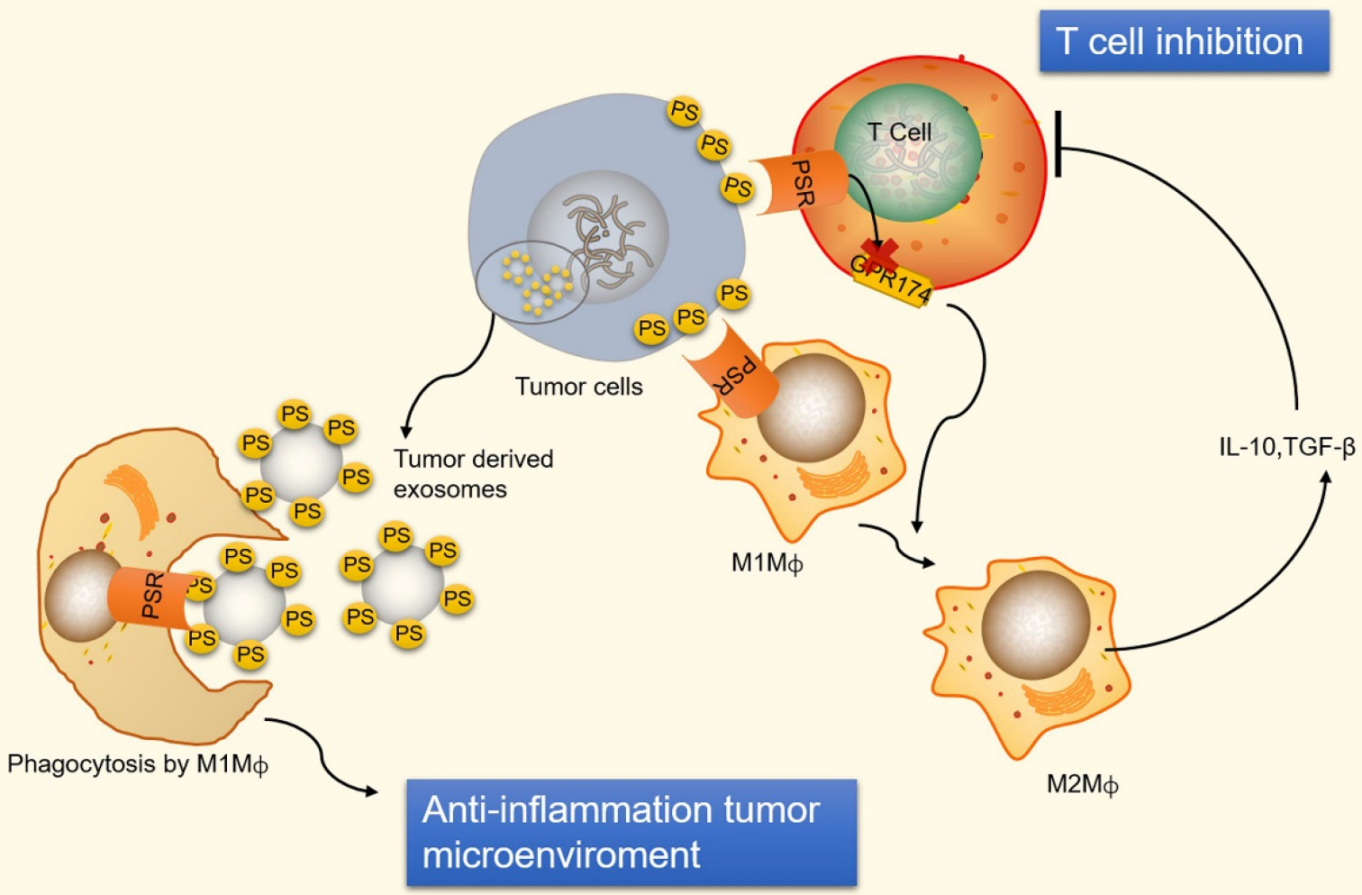
** An illustration of PS exposure on tumor cells and vesicles inducing immune suppression.** PS exposure on tumor cells induces immune suppression by ligation of PS to receptors on macrophages and T cells. PS binds to PSR on macrophages to promote maturation of M2-like macrophages, which are able to secrete the anti-inflammatory cytokines IL-10 and TGF-β. IL-10 and TGF-β are immunosuppressive cytokines that inhibit T cell activation. Additionally, ligation of PS to receptors on T cells inhibits T cell activation *via* GPR174-mediated M2 macrophage maturation. Conversely, PS exposed on tumor-derived microvesicles (externalized PS on microvesicles) increase the removal of apoptotic cells *via* phagocytes to prevent an undesirable inflammatory response and maintain an anti-inflammatory status in tumor microenvironments. GPR174, G protein-coupled receptor 174; M1Mф, M1-like macrophages; M2Mф, M2-like macrophages.

Immunosuppression and inflammation contribute to the creation of an environment that facilitates tumor growth and proliferation 118-120. Tumor cells can escape immune system surveillance by disrupting any step of T cell activation, or some tumor cells escape immune elimination by recruiting immunosuppressive leukocytes and orchestrating an antitumor microenvironment 121. PS is naturally exposed on tumor cells, the immature tumor vasculature and tumor-derived microvesicles 122-124. In addition, radiation therapy also causes an increase in the expression of PS on the surface of viable immune infiltrates in mouse B16 melanoma 125. The PS exposure on the surface of tumor cells prevents immune reaction by ligation of PS to receptors present on dendritic cells, macrophages and T cells 126. The ligation of PS to receptors on macrophages promotes macrophage polarization from a proinflammatory M1-like phenotype towards a protumor M2-like phenotype, allowing the secretion of the anti-inflammatory cytokines interleukin-10 (IL-10) and TGF-β 127. IL-10 and TGF-β are immunosuppressive cytokines that inhibit T cell activation by inhibiting tumor antigen presentation by dendritic cells and inducing regulatory T cells 128, 129 (**Figure 3**). Additionally, ligation of PS to receptors on T cells inhibits T cell activation *via* G protein-coupled receptor 174 (GPR174). GPR174-deficient Treg cells also promote the polarization of macrophages towards M2 and elevated expression of IL-10 130 (**Figure 3**).

Conversely, PS is exposed on tumor-derived microvesicles, and microvesicles externalizing PS show increased removal of apoptotic cells *via* phagocytes, preventing an undesirable inflammatory response and maintaining an anti-inflammatory status in tumor microenvironments (**Figure 3**). Indeed, PS exposure on microvesicles (exosomes) derived from patient tumor samples has been shown to suppress the activation of T cell responses 131. However, PS exposure on tumor cells also favors antitumor effects by mediating long-lived inflammation. A recent study has shown that the exposure of PS on tumor cells is necessary for IFNγ and IL-12 binding and the conversion of transient cytokine stimuli into long-lived inflammation, thus mitigating immunosuppressive functions 132. Therefore, a complex mechanism underlies the immune response to PS exposure and its immune suppressive effects in tumor microenvironments.

### PS and PS species as an imaging tool and biomarkers for cancer, respectively

Tumor cells expose PS on their surface, and therefore, methods for labeling PS are useful for tumor imaging 133. A liposomal nanoprobe PGN-L-IO/DiR, which binds specifically to PS and is subsequently internalized into cells, was shown to be a good imaging contrast agent for mice bearing MDA-MB231 breast tumors 134. Similarly, using a cyanine dye, indocyanine green, bound to PS antibodies helped to track and image apoptosis in triple-negative breast cancer cells 135, facilitating an effective treatment plan. In addition, PS-recognizing peptide 1 (PSP1) selectively binds to apoptosis-induced tumors in a radiation dose-dependent manner, which allows it to be used as an index probe to determine whether to continue radiation therapy in colorectal cancer 136.

Moreover, PS species can be biomarkers for cancer diagnosis. A clinical study conducted in 15 prostate cancer patients and 13 healthy controls examined the 36 most abundant lipid species. The results showed that a certain species of PS, PS (18:1/18:1), showed high significance between control and prostate cancer patients; furthermore, combinations of PS (18:1/18:1), lactosylceramide (d18:1/16:0) and PS (18:0/18:2) distinguished the two groups with 93% sensitivity and 100% specificity 137. More recently, a study compared lipids in surgical aerosols between tumor and adjacent normal tissues in lung cancer patients. Overexpression of PS (34:2), phosphatidylcholine (36:4), and triacylglycerol (46:2) and decreased phosphatidylcholine (34:3) were observed in cancerous aerosols 138, indicating that PS combined with other indices could be potential diagnostic biomarkers for lung cancer. In addition, species of PS may correlate with cancer proliferation and progression. The fatty acyl chain of PS has been shown to determine signal transduction efficiency. Studies have shown that nanoclusters of K-Ras, an important transduction signal, colocalize with markers of PS 139, and these nanoclusters are associated with PS species with one saturated and one unsaturated fatty acyl group (PS 18:0/18:1 or PS 16:0/18:1) 140. K-Ras is an isoform of Ras GTPase, in which mutations are commonly found in many cancers.

## PS binding molecules and cancer therapy

As we discussed above, PS exposure on tumor and immune cells regulates immune responses in tumor microenvironments. Blocking PS on tumor cells restricts phagocytosis and T cell-mediated killing. Thus, targeting PS is considered a promising strategy for cancer therapy. Both preclinical and early phase clinical trials using PS targeting agents, including monoclonal antibodies, antibody-drug conjugations, liposomal carriers and natural products, have shown potential antitumor activities. Synergistic effects of PS targeting antibodies in combination with traditional cancer drugs have been observed in clinical trials. The therapeutic strategy of blocking PS is based mainly on two methods: 1) Disrupting PS on tumor cells. 2) Disrupting receptor signaling by targeting PS receptors.

### Disrupting direct PS binding in cancer therapy

#### 2aG4 and bavituximab

**Figure 4:**
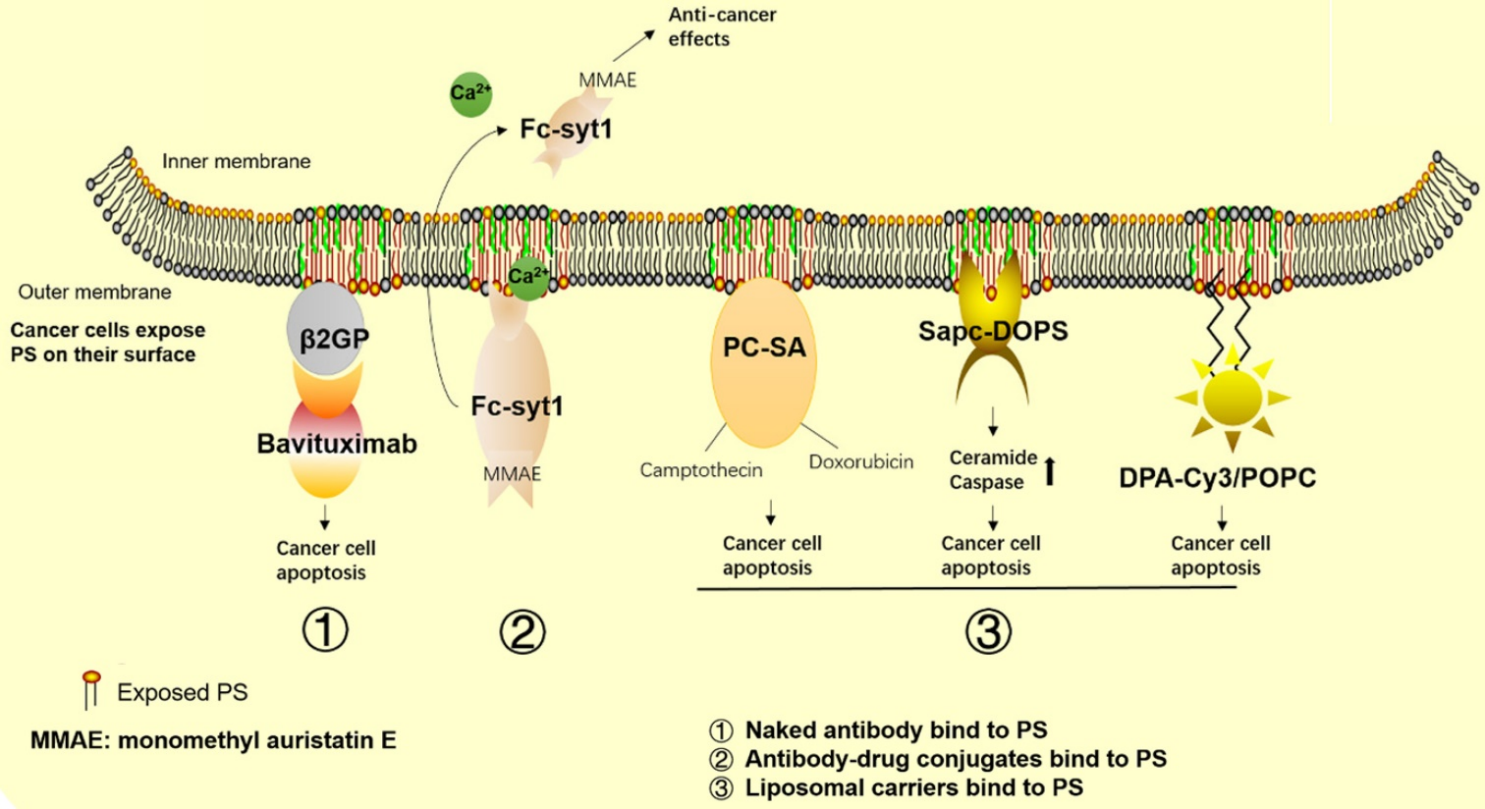
** Cancer therapeutics related to PS.** The graph shows the approved and promising drugs designed to target PS for cancer therapy. 1) Naked antibodies bind to PS. 2) Antibody-drug conjugates bind to PS. 3) Liposomal carriers bind to PS.

In recent years, a number of agents targeting PS have been developed for cancer therapy. Among these agents, bavituximab, a human-mouse chimeric monoclonal antibody that binds to PS indirectly by linking to β2GP1 with high affinity (**Figure 4**), has been the most studied. Mechanistic studies have shown that bavituximab blocks PS and exerts its antitumor immunity effects by promoting M1 macrophage maturation, as well as by inducing cellular cytotoxicity of tumor-associated endothelial cells. In experimental studies, 2aG4, the murine version of bavituximab, has been shown to exert a superior therapeutic effect in combination with the hepatocellular carcinoma therapy drug sorafenib compared with its use alone 141. Further mechanistic studies have shown increased PS exposure in response to sorafenib treatment in the tumor vasculature, while 2aG4 targets PS and significantly increases the amount of M1 macrophages, exerting its antitumor effects by secreting TNF-α, IL-12 and promoting the Th1 response. These findings indicate that 2aG4 targets PS to regulate the immune function of cells. In addition, the effects of transforming M2 to M1 macrophages are observed in prostate tumor-bearing mice by treatment with 2aG4 in combination with docetaxel 142. A recent study also showed that a manmade immunocytokine, 2aG4-IL2, which genetically links IL-2 to 2aG4, blocks PS induced immunosuppression in lung cancer. The vaccine was generated by coating tumor cells with 2aG4-IL2, and it reduced the incidence and number of spontaneous lung metastases 143. In addition, the combination of 2aG4 and APR-246, which is a small molecule drug that restores p53 function, effectively inhibited tumor growth in advanced hormone-dependent breast cancer 144.

In clinical trials, bavituximab is used as a single agent or in combination with other traditional therapies in the treatment of lung cancer, hepatocellular carcinoma, breast cancer, pancreatic cancer, and solid tumors. By summarizing all the clinical results (**Table 1**), we found that as a monotherapy or in combination with chemotherapy or radiation therapy, bavituximab shows minimal side effects in the treatment of patients with lung cancer, hepatocellular carcinoma, breast cancer, solid tumors and rectal adenocarcinoma 141, 145-148. In phase Ⅰb and Ⅱ clinical trials, as a front line drug, bavituximab showed an overall response rate (ORR) of 28% and a progression-free survival rate (PFR) of 4.8% in combination with carboplatin and pemetrexed, and an ORR of 40.8% and PFR of 6.0% in combination with carboplatin and paclitaxel in non-small cell lung cancer therapy 149, 150. As a second-line drug, bavituximab also showed good ORRs and PFRs in combination with docetaxel in non-small cell lung cancer treatment 151. Similar results were observed in phase I and II clinical trials in the treatment of metastatic breast cancer; as a front-line drug, bavituximab combined with paclitaxel showed an ORR of 85% and a PFR of 4.8% in HER2-negative breast cancer 147. As a second-line drug, bavituximab combined with docetaxel showed an ORR of 60.9% and a PFR of 7.4%. In other phase II trials, bavituximab also showed good therapeutic results in the treatment of hepatocellular carcinoma in combination with sorafenib 152 and in the treatment of pancreatic cancers in combination with gemcitabine 153. However, a recent phase III clinical trial in non-small cell lung cancer patients showed that bavituximab was not sufficient to improve overall survival compared to treatment with docetaxel alone 154. This result is frustrating for the pharmaceutical company developing bavituximab; however, a retrospective case study analysis showed that this failure was due to poor quality data in phase II clinical trials and commercial consideration, so the phase III clinical trials should have been stopped earlier 155. To date, there are no other phase III clinical trial results for bavituximab in other cancer treatments, and whether this drug can be used in cancer treatments outside of non-small cell lung cancer or should be abandoned is still unknown. Concurrently, other strategies targeting PS are in development for tumor suppression.

### Antibody-drug conjugates

Antibody-drug conjugates are another branch of developing PS-targeting drugs. Antibody-drug conjugates combine a PS-targeting antibody to a cytotoxic drug to exert tumor killing effects. Experimental studies have shown that some antibody-drug conjugates have better tumor suppressive effects than “naked” antibodies. For example, Fc-Syt1 is a PS-targeting agent generated by fusing PS-binding domains to a human IgG1-derived Fc fragment C2A. Use of a Fc-Syt1 conjugated to monomethyl auristatin E, a cytotoxic drug, showed good antitumor effects in mouse breast and prostate cancer models 156. In addition, recent researchers have developed a new method in which a PS binding peptide, PSBP-6, is conjugated to pH-sensitive mixed micelles (PEG-PDLLA and PEG-PHIS) and then loaded onto the chemotherapeutic agent paclitaxel (PTX) in prepared micelles. These pH sensitive micelles represent an acid triggered drug release system that is suitable for the acidic tumor environment. An *in vivo* study showed that the conjugated agents had improved cytotoxicity and uptake by tumor cells, as well as accumulation at tumor sites 157.

Moreover, fusion proteins consisting of L-methionase linked to human Annexin-V, an antibody with high affinity for PS, have shown good effects in tumor cell killing compared with L-methionase with no fusion protein present 158. Moreover, the fusion protein shows almost no effects on normal cells, indicating that this strategy is a promising approach for new drug development.

### Liposomal carriers

Liposomes are used as drug carriers because of their drug protecting and specific targeting characteristics. Targeting PS is a main antitumor mechanism of some liposomal carriers. Those liposomal carriers bind to PS on tumor cells and exert antitumor effects either by metabolic interference or synergistic effects with drug loading.

SapC-DOPS is a protein/lipid nanovesicle composed of the sphingolipid-activating protein Saposin C (SapC), which functions to catabolize glycosphingolipids and dioleoylphosphatidylserine (DOPS). SapC-DOPS selectively binds to PS on cancer cells and induces cell apoptosis *via* ceramide accumulation and caspase activation 159 (Figure 4). Research groups have used SapC-DOPS to treat different cancer cells, such as brain tumor cells 160, skin cancer cell lines 161 and pancreatic cancer cells 162. The results showed that SapC-DOPS has good antitumor effects in a number of primary and metastatic tumors. The same group later found that during irradiation therapy, cells with high levels of surface PS had a higher survival rate, which inversely correlated with sensitivity to radiation therapy and some chemotherapy. However, these cells are sensitive to SapC-DOPS treatment, suggesting that SapC-DOPS can be used as a combination therapy for cancer cells with high PS exposure during radiation therapy 163. A recent review focusing on cancer therapy treatments with SapC-DOPS is available 164.

In addition, phosphatidylcholine-stearylamine (PC-SA) is a cationic liposomal carrier that specifically binds to PS on cancer cells and tumors. A preclinical study showed that PC-SA has anticancer effects as a single agent and has higher efficacy when loaded with traditional antitumor drugs (**Figure 4**). In this study, using PC-SA alone or the anticancer drugs camptothecin and/or doxorubicin entrapped in PC-SA liposomes inhibited tumor growth and decreased tumor microvasculature formation in three tumor models. PC-SA enhances the half-life of antitumor drugs and shows no signs of obvious toxicity to other organs, suggesting its potential as a new drug or drug carrier for cancer combination therapy 165.

DPA-Cy3 is a lipid-soluble zinc(II)-bis-dipicolylamine derivative that contains the fluorophore cyanine 3 (Cy3) and two 22-carbon chains that can be anchored into liposomal membrane bilayers. Use of DPA-CY3 and 1-palmitoyl-2-oleoyl-sn-glycero-3-phosphocholine (POPC) liposomes resulted in selective binding to PS-enriched cancer cells. That study demonstrated that DPA-CY3/POPC could exert antitumor effects without any drugs loaded. DPA-CY3/POPC prefers to bind to the surface of breast cancer cells (MCF-7) versus noncancer cells (MCF-12A) due to the different levels of PS exposure. Additionally, internalization of DPA-CY3/POPC by MCF-7 is more stable than that by MCF-12A, and thus, the cytotoxic effects to tumor cells are more robust in tumor cells compared to normal cells 166.

### Natural plant extracts targeting PS

It is worth noting that some natural products also show anticancer effects by targeting PS. For example, Chalepin is a compound extracted from the plant *Ruta angustifolia* that has been shown to exert cytotoxic activity against breast cancer cells but not normal cells. Mechanistic studies have shown that the induction of apoptosis by Chalepin is associated with PS externalization 167.

### Targeting PS receptors and cancer therapy

As discussed in the “PS receptors” section, previous research has shown that PS receptors participate in the progression of tumor cells and control immune responses in the tumor microenvironment. Agents targeting TIM and TAM receptors have been developed for cancer treatment. TIM-3 antibody suppresses tumor growth *via* T cell regulation, especially in combination with anti-PD-1 drugs, in the treatment of fibrosarcomas 168. Blockade of TIM-3 enhances the antitumor immune response in head and neck cancer 169. Not surprisingly, a few pharmaceutical companies are now developing anti-TIM-3 antibodies as new antitumor agents, such as TSR-022 (NCT02817633; Tesaro), MBG543 (NCT02608268; Novartis), BMS-986258 (NCT03446040; MBS), and LY3321367 (NCT03099109; Eli Lilly and Company), which are all in phase I/II clinical trials 170-172. Most of those trials have not yet provided results. However, some results from the TSR-022 phase I clinical trial were released at the 2018 annual meeting of the Society for Immunotherapy of Cancer (SITC). The results showed that the combination of TSR-022 with PD-1 antibody showed good tolerance in both non-small cell lung cancer and melanoma patients, and a high dose of TSR-022 (300 mg) showed observed activity, with an ORR of 15% (3/20) and 40% stable disease (8/20) 173. The results showed the promise of anti-TIM-3 drugs in cancer treatment.

Agents that target TAM have also attracted much attention. TAM is abnormally expressed in various cancer cells, including acute myeloid leukemia, gastric and colorectal adenocarcinomas, non-small cell lung, breast and prostate cancers, and it is considered an oncogene. In this case, PS receptor inhibitors were developed to help enhance the innate immune response and kill cancer cells. Multiple drugs are being developed to selectively or nonselectively target TAM receptors. For example, Sitravatinib is a multikinase inhibitor that targets all three TAM receptors, and the combination of Sitravatinib with the PD-1 inhibitor Nivolumab is currently in phase Ⅱ (NCT02954991) and phase Ⅲ (NCT03906071) clinical trials for non-small cell lung cancer treatment. Additionally, the use of Sitravatinib for other cancer treatments, such as in combination with Tislelizumab for advanced or metastatic hepatocellular carcinoma (HCC) or gastric/gastroesophageal junction cancer (GC/GEJC) (NCT03941873), for advanced solid tumors (NCT03666143), and in combination with Nivolumab for advanced or metastatic kidney cancer (NCT03015740), urothelial carcinoma (NCT03606174) and clear cell renal cell carcinoma (NCT03680521), are in the recruiting phase for phase I or phase II clinical trials. Additionally, some agents that were initially not designed for TAM targeting were found to suppress TAM concurrently with their main targets. Those drugs, such as bosutinib 175, 176, crizotinib 177, 178, and cabozantinib 179, 180, have been approved for clinical use 174 as nonselective drugs. Moreover, many drugs have been developed to selectively target one of the TAM receptors, including selective AXL inhibitors (R428, TP0903, BMS777607 and NPS1304), selective MerTk inhibitors (MRX2843, UNC2025, UNC3133 and ONO7475) and Tyro3 inhibitors (Pfizer compounds 11, 12, 19, 21 and KRCT-6j) 91. Most of these agents have demonstrated synergistic effects to overcome the resistance of classical antitumor drugs. BMS777607 recovers Sunitinib sensitivity in advanced renal cell carcinoma 181, and NPS1034 restores gefitinib or erlotinib sensitivity in an EGFR-resistant lung cancer cell model 182. These selective TAM receptor drugs are designed to lower the side effects of nonselective TAM receptor drugs. Most of these agents do not specifically target TAM but also suppress other kinase families. For example, BMS777607 suppresses c-MET and AXL, and MRX2843 suppresses MerTK and Flit.

It should be noted that targeting TIM does not fully represent inhibition of PS because TIM can bind to other proteins; for example, TIM-3 has been identified to interact with galectin-9 (gal-9) and high-mobility group protein box 1 (HMGB-1) 183, 184, which also participate in immune responses. Moreover, conflicting results were reported in studies of TAM receptor targeting. MerTK-deficient tumors show increase leukocyte proliferation and higher infiltration of CD8^+^ T lymphocytes in a mouse model 185. Additionally, MerTK deficiency shows better tumor control after radiotherapy in colorectal and pancreatic adenocarcinoma mice 186. However, another study showed that MerTK and AXL-deficient mice exhibit exacerbated tumor growth and inflammation associated cancer 186. In addition, a recent study showed that MerTK expression on CD8^+^ T cells improves tumor-infiltrating lymphocyte expansion 187. The discrepancy between different studies may result from the different tumor types and PS exposure levels studied, but these results provide a hint that blindly inhibiting TAM, at least in the case of MerTK inhibitory agents, should be used with caution due to its potential for inducing inhibition of tumor killing.

## PS exposure and CD47 for cancer therapy

PS exposure on the cell surface provides a phagocytic signal for macrophages, while CD47 expression on cells inhibits phagocytosis 124. CD47 is widely expressed on all cells but has high expression levels on various tumor cells 188. CD47 is a ligand of signal regulatory protein-α (SIRPα), a protein expressed on macrophages and dendritic cells. Upon binding CD47, SIRPα initiates a signaling cascade that results in the inhibition of phagocytosis 189, through which tumor cells can escape from immune surveillance of macrophages and T cells 190. In erythrocytes, CD47 ligation induces PS expression as part of a death pathway 191, suggesting that CD47 could affect PS exposure. Indeed, knockout of CDC50A, a subunit of ATP11C that participates in PS flipping, increases tumor-associated macrophages and enhances the effect of anti-CD47 blockade on limiting tumor growth 192. Knockout of CDC50A also increases PS exposure on Jurkat cells, which may affect T cell function, as we mentioned earlier. Thus, blockade of CD47 inhibits phagocytosis, however, PS exposure has been utilized to target tumor cells for macrophage clearance. In clinical trials, the combination of anti-CD47 agents has shown synergistic antitumor effects. The anti-CD47 inhibitor 5F9 combined with rituximab (anti-CD20 antibody) has shown a good response with minimal side effects in patients with aggressive and indolent lymphoma 193.

## Perspective and Conclusion

Currently, immunotherapy that enhances T cell immunity has become a hot research area. PS exposure is known to be an immune suppressor in tumor microenvironments. In general, agents that directly or indirectly target PS rescue immune suppression, enhance antitumor drug activity and are accompanied by good tolerance, suggesting that PS is promising as a new drug for mono- or polytherapy for cancer. However, we notice that some risks arise during treatment with this kind of drug. First, inhibition of PS receptors, such as MerTk on T cells, may reduce tumor killing effects. Second, PS exposure on the cell surface provides a phagocytic signal for macrophages, and some immune therapies have utilized this mechanism to target tumor cells for macrophage clearance, such as CD47 inhibitors; thus, using anti-PS agents may modulate those drug therapy effects. Third, anti-PS therapy is mainly utilized in combination with other cancer drugs, and the safety and unknown side effects, such as the risk of developing thromboembolic events, is largely unknown. Therefore, although targeting PS shows promise in cancer therapy, many obstacles must be overcome for its successful application in the clinic. Ideally, agents that specifically target PS on tumor cells but do not affect PS on normal cells, and agents that sense the quantity of PS exposure when in combination with other cancer drugs, merit further investigation. Future research could focus on these obstacles and challenges to develop new kinds of drugs.

## Figures and Tables

**Table 1:** Summary of clinical trials evaluating the combination of PS-targeting antibodies with chemotherapy or radiation

Clinical trial phase	Tumor type	Drug name targeting PS	N	Duration (months)	Chemotherapy orradiation combination	Tumor growth inhibition	Side effects	Reference
Phase Ib	Front line-advanced non-small-cell lung cancer	Bavituximab (0.3, 1 or 3 mg/kg)	26	Once every 3 weeks for 6 cycles	Carboplatin pemetrexed	RR, 28% PFS, 4.8 OS, 12.2	Well tolerate	149
Phase II	Front line-Advanced non-small-cell lung cancer	Bavituximab (3 mg/kg)	49	Once every 3 weeks for 6 cycles then monotherapy	Carboplatin Paclitaxel	RR, 40.8% PFS, 6.0 OS,12.4	40.8%	150
Phase II	Second line-Advanced nonsquanous non-small-cell lung cancer	Bavituximab (1 or 3 mg/kg) or placebo	121	Once every 3 weeks for 6 cycles	Docetaxel	1mg-RR,11.3%; PFS, 4.5 3mg-RR,17.1%; PFS, 4.5	Well tolerate	151
Phase III	Second line-Advanced non-small-cell lung cancer	Bavituximab (3 mg/kg) or placebo	597	Once a week	Docetaxel	Not superior to Docetaxel monotherapy	Well tolerate	154
Phase I	Front line-HER-2 negative metastatic breast cancer	Bavituximab (3 mg/kg)	14	Once every 4 weeks for 4 cycles	Paclitaxel	RR, 85%; PFS, 7.3	--	147
Phase II	Second line-advanced or metastatic breast cancer.	Bavituximab (3 mg/kg)	46	Once a week for first 3 weeks in a 28-day cycle of 6 cycles	Docetaxel	RR, 60.9%; PFR, 7.4	Well tolerate	194
Phase I	Preoperative treatment of Rectal adenocarcinoma	Bavituximab (0.3, 1 or 3 mg/kg)	14	Once a week for 8 weeks	Radiation and capecitabine	--	Well tolerate	146
Phase I	Hepatocellular carcinoma	Bavituximab (0.3, 1 or 3 mg/kg)	9	Once a week for 8 weeks	Sorafenib or monotherapy	--	Well tolerate	195
Phase II	Front line-advanced hepatocellular carcinoma	Bavituximab (3 mg/kg)	38	Once a week	Sorafenib	PFS, 6.7; OS, 6.2		152
Phase I	Advanced solid tumors	Bavituximab (0.3, 1 or 3 mg/kg)	26	Once a week for 8 weeks	--	--	Well tolerate	145
Phase Ib	Front line-advanced solid tumors	Bavituximab (3 mg/kg)	14	Once a week for 8 weeks	Docetaxel or gemcitabine or paclitaxel plus carboplatin	--	Well tolerate	148
Phase II	Front line-Stage IV Pancreatic Cancer	Bavituximab (3 mg/kg) vs Gemcitabine alone	70	Once a week for first 3 weeks in a 28-day cycle	Gemcitabine	ORR, 28.1 vs 12.9%; OS, 5.6 vs 5.2 months	Well tolerate	153
Phase II	advanced gastric or gastroesophageal junction cancer	Bavituximab	80		Pembrolizumab	Still in progress		

OS, median overall survival time; ORR, overall response rate; PFR, Progression-free survival rate.
